# Three-Dimensional Gene Regulation Network in Glioblastoma Ferroptosis

**DOI:** 10.3390/ijms241914945

**Published:** 2023-10-06

**Authors:** Man Liu, Wenbin Wang, Han Zhang, Jinfang Bi, Baoying Zhang, Tengfei Shi, Guangsong Su, Yaoqiang Zheng, Sibo Fan, Xiaofeng Huang, Bohan Chen, Yingjie Song, Zhongfang Zhao, Jiandang Shi, Peng Li, Wange Lu, Lei Zhang

**Affiliations:** State Key Laboratory of Medicinal Chemical Biology, Frontiers Science Center for Cell Responses, College of Life Sciences, Nankai University, Tianjin 300071, China; 1120160376@mail.nankai.edu.cn (M.L.); 2120181086@mail.nankai.edu.cn (W.W.); 2120211193@mail.nankai.edu.cn (H.Z.); 1120180423@mail.nankai.edu.cn (J.B.); 2120201068@mail.nankai.edu.cn (B.Z.); 1120190496@mail.nankai.edu.cn (T.S.); 819085@nankai.edu.cn (G.S.); 1120190507@nankai.edu.cn (Y.Z.); 2120211186@nankai.edu.cn (S.F.); 2120221406@nankai.edu.cn (X.H.); 1120200511@mail.nankai.edu.cn (B.C.); 1120210558@mail.nankai.edu.cn (Y.S.); zhfzhao@nankai.edu.cn (Z.Z.); shijd@nankai.edu.cn (J.S.); lipeng@nankai.edu.cn (P.L.)

**Keywords:** glioblastoma, ferroptosis, 3D chromatin structure, HiChIP, enhancer

## Abstract

Ferroptosis is an iron-dependent form of cell death, which is reported to be associated with glioma progression and drug sensitivity. Targeting ferroptosis is a potential therapeutic approach for glioma. However, the molecular mechanism of glioma cell ferroptosis is not clear. In this study, we profile the change of 3D chromatin structure in glioblastoma ferroptosis by using HiChIP and study the 3D gene regulation network in glioblastoma ferroptosis. A combination of an analysis of HiChIP and RNA-seq data suggests that change of chromatin loops mediated by 3D chromatin structure regulates gene expressions in glioblastoma ferroptosis. Genes that are regulated by 3D chromatin structures include genes that were reported to function in ferroptosis, like *HDM2* and *TXNRD1*. We propose a new regulatory mechanism governing glioblastoma cell ferroptosis by 3D chromatin structure.

## 1. Introduction

Glioma is a common intracranial primary tumor. About 80% of malignant primary brain tumors are glioblastoma (GBM), which is the most malignant glioma and has a high mortality rate [1,2]. Currently, the clinical treatment methods for glioma are limited, and patients’ median survival time is only 14–17 months [3]. Despite the fact that glioma therapeutic approaches have improved in recent years, the clinical effects and prognosis are still not satisfactory. Finding the accurate drug target is important, and problems such as drug resistance and immunosuppression in glioma treatment have not yet been fully solved. Tumor cell death is key in tumor therapy, and elucidating the molecular mechanisms of tumor cell death is important to the development and improvement of therapeutic approaches. However, the molecular mechanisms of glioma cell death remain unclear.

Ferroptosis is a newly defined form of cell death (2012), which is caused by iron-induced oxidative damage, the disruption of the cell membrane, and cell lysis [4,5]. Ferroptosis is related to oncogenesis and tumor development and is regulated by multiple cancer-associated signaling pathways [6]. Recent studies have shown that ferroptosis inhibits the growth of glioma cells, which is associated with the survival of patients and the clinical outcome of radiotherapy and chemotherapy [7], but the mechanisms underlying this are not clear.

The development of 3D genomic technologies reveals that gene regulatory elements regulate distal target genes’ expression through the 3D structure of chromatin, and the 3D gene regulatory network can regulate physiological and biochemical processes in cells [8,9,10]. Several studies reported that change in the 3D structure of chromatin regulates the expression of oncogenes and tumor suppressor genes and affects tumor initiation and development [10,11,12,13,14,15,16]. As an important physiological process that affects glioma initiation and progression, ferroptosis involves multiple important signaling pathways, and the related gene regulation may be associated with the change of the 3D chromatin architecture. In this study, H3K27ac HiChIP was performed to investigate the change of active enhancer-related 3D chromatin structures in glioblastoma cell ferroptosis. By combining an analysis of RNA-seq and ChIP-seq data, this study found a 3D enhancer-gene regulation network in glioblastoma cell ferroptosis.

## 2. Results

### 2.1. Distribution of Active Enhancers in Ferroptotic Glioblastoma Cells

**Figure 1:**
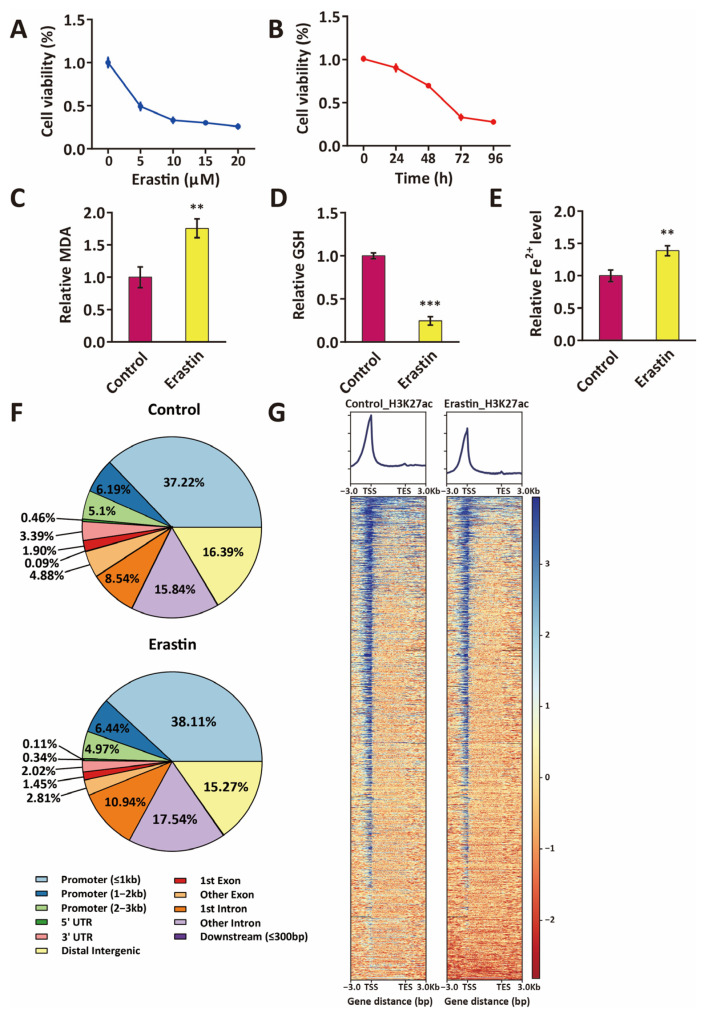
Distribution of active enhancers in ferroptotic glioblastoma cells. (**A**) Cell viability of U87MG treated with 0, 5, 10, 15, and 20 μM Erastin for 72 h, compared to control (blue line). Data represent means ± S.E.M. of three independent experiments. (**B**) Cell viability of U87MG that were treated with 10 μM Erastin for 0 h, 24 h, 48 h, 72 h, and 96 h, compared to control (red line). Data represent means ± S.E.M. of three independent experiments. (**C**–**E**) MDA assay, GSH assay, and ferrous iron assay indicate an increase of MDA (**C**), a decrease of GSH (**D**), and an accumulation of intracellular Fe^2+^ (**E**) in U87MG cells that were treated with 10 μM Erastin for 72 h. MDA, malondialdehyde; GSH, glutathione. Data represent means ± S.E.M. of three independent experiments. ** *p* < 0.01, *** *p* < 0.001, compared to control. (**F**) Pie chart showing the genome-wide distribution of active enhancer markers (H3K27ac) in U87MG and Erastin (10 μM, 72 h)-treated U87MG cells. (**G**) Heatmap showing H3K27ac ChIP-seq signal enrichment around TSS site in U87MG and Erastin-treated U87MG cells.

Erastin is a small molecular compound that inhibits the system Xc- and prevents the import of cystine, reducing glutathione (GSH) biosynthesis and glutathione peroxidase 4 (GPX4) activity and finally inducing cell death via ferroptosis [17]. To establish a model of Erastin-induced ferroptosis in glioblastoma cells, U87MG cells were treated for different concentrations of Erastin. Significant survival inhibition of U87MG was detected by MTS assay under the treatment of Erastin, and higher concentrations or longer treatment times of Erastin induced a more significant decrease in cell viability (Figure 1A,B and Figure S1A,B). U87MG cell survival inhibition reached lower than 50% after 72 h of treatment with 10 μM Erastin (Figure 1A and Figure S1A). Malondialdehyde (MDA) is the product of polyunsaturated fatty acid peroxidation. The level of MDA in cells indicates the degree of cellular oxidation, which can be used to quantify ferroptosis. A significant increase of intracellular MDA levels in U87MG glioblastoma cells was detected after Erastin treatment (10 μM, 72 h) compared to control (Figure 1C and Figure S1C). The depletion of glutathione (GSH) is associated with an increase in cell ferroptosis, and significant GSH depletion was shown in the Erastin (10 μM, 72 h)-treated U87MG cells (Figure 1D and Figure S1D). In addition, the accumulation of intracellular Fe^2+^ is a marker of increased ferroptosis, and the level of Fe^2+^ significantly increased in Erastin (10 μM, 72 h)-treated U87MG cells compared to control (Figure 1E and Figure S1E). These data suggest significant ferroptosis in U87MG cells treated with 10 μM Erastin for 72 h.

H3K27ac is known as a marker of active enhancers in mammals [18,19]. Enrichment of H3K27ac modification is associated with the upregulation of gene expression. H3K27ac ChIP-seq was performed in control glioblastoma cells and glioblastoma cells that were treated with Erastin (10 μM, 72 h) to investigate the distribution of active enhancers in control glioblastoma cells and ferroptotic glioblastoma cells. In both control glioblastoma cells and ferroptotic glioblastoma cells, ~50% of all H3K27ac peaks are at promoters, ~40% are at non-coding regions, such as intergenic regions and introns, and ~5% appeared in exon regions (Figure 1F). No significant global changes in H3K27ac enrichment were detected in ferroptotic glioblastoma cells relative to control cells (Figure 1F). H3K27ac peaks are mainly enriched near the transcriptional start sites of the genes in both control glioblastoma cells and ferroptotic glioblastoma cells (Figure 1G). 

### 2.2. HiChIP Identifies the Change of 3D Chromatin Structure in Glioblastoma Cell Ferroptosis

**Figure 2:**
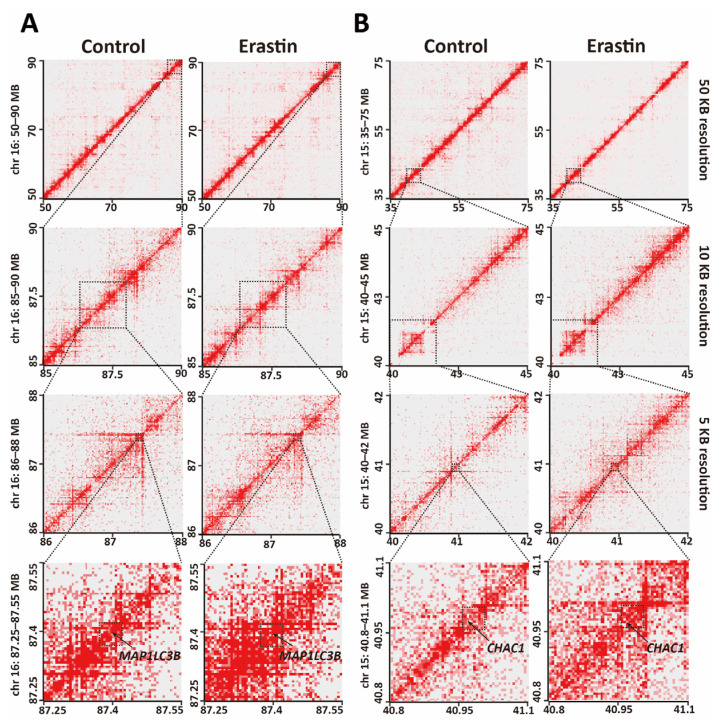
Changes in 3D chromatin structures in glioblastoma cell ferroptosis. (**A**,**B**) HiChIP heatmaps at a resolution of 50, 10, and 5 kb around *MAP1LC3B* (**A**) and *CHAC1* (**B**) loci. Dotted squares indicate regions with increased chromatin interactions in Erastin (10 μM, 72 h)-treated U87MG glioblastoma cells compared to control.

To investigate functions of 3D genome structure in glioblastoma cell ferroptosis, H3K27ac HiChIP was performed to identify enhancer-related 3D chromatin structures in control glioblastoma cells and ferroptotic glioblastoma cells. HiChIP identified 66172 and 30722 high-confidence, reproducible chromatin loops in control glioblastoma cells and Erastin (10 μM, 72 h)-treated ferroptotic glioblastoma cells, respectively. HiChIP data indicated the change of 3D chromatin structure in ferroptotic glioblastoma cells compared to control (Figure 2A,B). *MAP1LC3B* and *CHAC1* were reported to function in ferroptosis [20,21,22,23]. 

To determine whether the change of 3D chromatin structures induced a transcription change of genes in ferroptosis, RNA-seq was performed in U87MG cells and Erastin (10 μM, 72 h)-treated U87MG cells to detect the transcriptome change in ferroptotic glioblastoma cells. HiChIP data indicated increased chromatin interactions near *MAP1LC3B* and *CHAC1* loci in ferroptotic glioblastoma cells.

**Figure 3:**
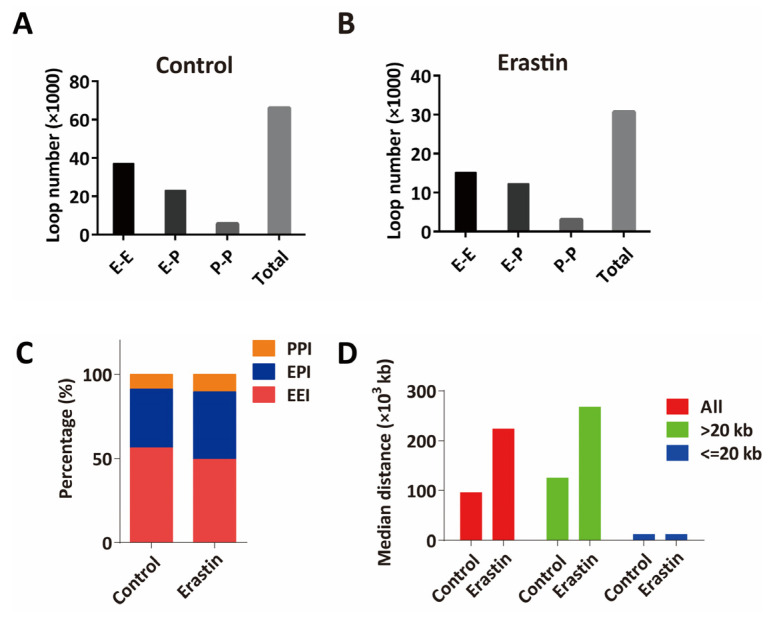
HiChIP identifies the change of 3D chromatin structure in glioblastoma cell ferroptosis. (**A**,**B**) The numbers of EEI, EPI, and PPI in control U87MG cells (**A**) and Erastin (10 μM, 72 h)-treated U87MG cells (**B**). (**C**) Percentages of EEI, EPI, and PPI in control U87MG cells and Erastin (10 μM, 72 h)-treated U87MG cells. (**D**) Median distances of EEI, EPI, and PPI in control U87MG cells and Erastin (10 μM, 72 h)-treated U87MG cells. EEI: enhancer–enhancer interaction, EPI: enhancer–promoter interaction, PPI: promoter–promoter interaction.

Chromatin loops that are identified by H3K27ac HiChIP include enhancer–enhancer interactions (EEI), enhancer–promoter interactions (EPI), and promoter–promoter interactions (PPI) (Figure 3A,B). Among all enhancer and promoter interaction networks in control U87MG glioblastoma cells, 56.2% were EEI, 35.1% were EPI, and 8.7% were PPI, while in Erastin (10 μM, 72 h)-treated U87MG glioblastoma cells, PPI and EPI increased to 10.3% and 40.1%, respectively, and EEI decreased to 49.6% (Figure 3C). Moreover, the median length of chromatin interaction loops was longer in Erastin (10 μM, 72 h)-treated U87MG glioblastoma cells compared to control, and more long-range chromatin interactions were detected in Erastin-treated U87MG cells (Figure 3D), suggesting global alterations of 3D chromatin structure in ferroptosis. 

### 2.3. Transcriptome Change in Ferroptotic Glioblastoma Cells Is Associated with Changes of 3D Chromatin Structure

**Figure 4:**
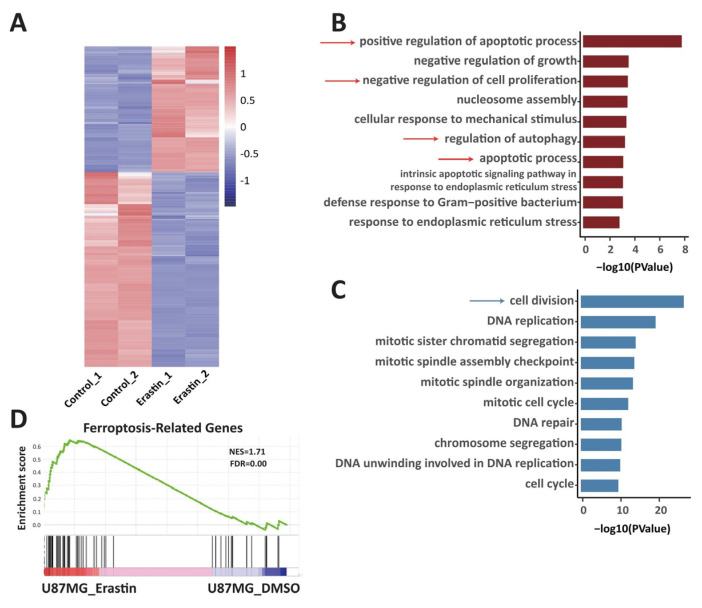
Transcriptome change in ferroptotic glioblastoma cells. (**A**) Comparison of gene expression in control U87MG cells (Control_1 and Control_2) and Erastin (10 μM, 72 h)-treated U87MG cells (Erastin_1 and Erastin_2). Heatmap shows clustering of differentially expressed genes in control U87MG cells and Erastin (10 μM, 72 h)-treated U87MG cells. (**B**) GO analysis of genes significantly upregulated in Erastin (10 μM, 72 h)-treated U87MG cells compared to control U87MG cells (log_2_FC > 1; *p_adj_* < 0.05) (dark red color). (**C**) GO analysis of genes significantly downregulated in Erastin (10 μM, 72 h)-treated U87MG cells compared to control U87MG cells (log_2_FC ≤ −1 and *p_adj_* < 0.05) (blue color). (**D**) Gene Set Enrichment Analysis (GSEA) of genes differentially expressed in control U87MG cells V.S. Erastin (10 μM, 72 h)-treated U87MG cells among genes associated with ferroptosis. The green curve represents the enrichment score curve in GSEA.

RNA-seq data indicated 917 genes were significantly upregulated (log_2_FC > 1; *p_adj_* < 0.05) and 1422 genes were significantly downregulated (log_2_FC ≤ −1; *p_adj_* < 0.05) in ferroptotic cells compared to control (Figure 4A). Gene ontology (GO) analysis of significantly upregulated gene revealed that positive regulation of apoptotic process, apoptotic process, regulation of autophagy and negative regulation of cell proliferation-related biological processes were enriched in ferroptotic glioblastoma cells (Figure 4B). These cell signaling pathways or biological processes are associated with ferroptosis [24,25,26]. GO analysis of significantly downregulated genes (log_2_FC ≤ −1, *p_adj_* < 0.05) showed that the top pathway altered was cell division (Figure 4C). In addition, the Gene Set Enrichment Analysis (GSEA) indicated that the ferroptosis-related genes [27] are highly enriched in Erastin (10 μM, 72 h)-treated U87MG cells (Figure 4D). 

**Figure 5:**
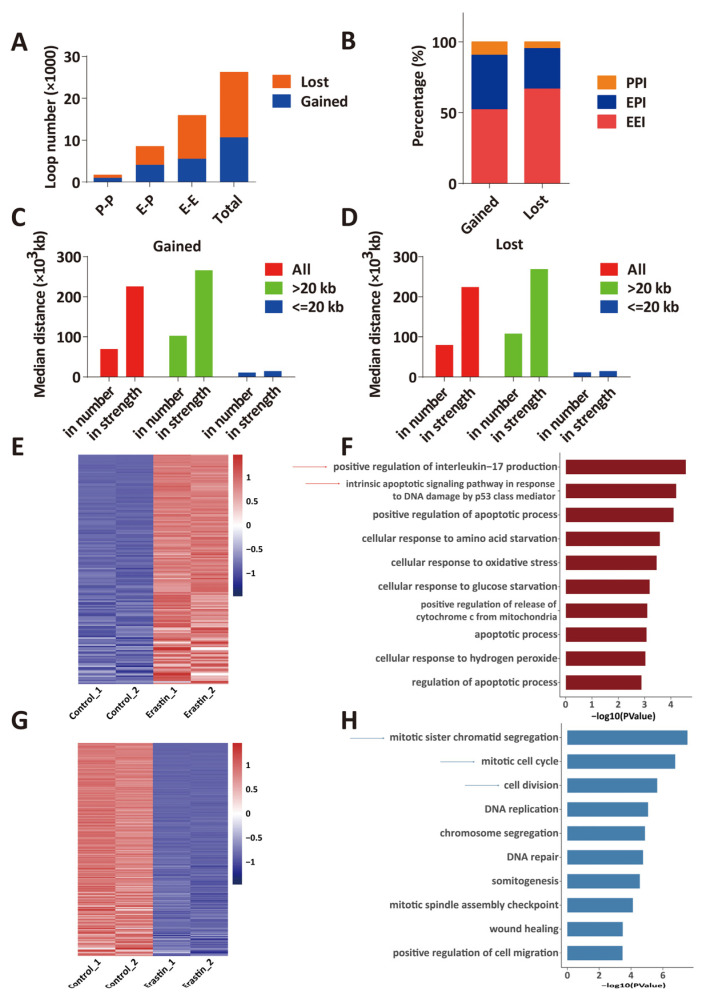
Transcriptome change in ferroptotic glioblastoma cells is associated with changes in 3D chromatin structures. (**A**) The numbers of EEI, EPI, and PPI of “gained” and “lost” chromatin loops in U87MG cells that were treated with Erastin (10 μM, 72 h). (**B**) Percentages of EEI, EPI, and PPI in all “gained” and “lost” loops in U87MG cells that were treated with Erastin (10 μM, 72 h). (**C**) Median distances of “gained” chromatin loops in U87MG cells that were treated with Erastin (10 μM, 72 h). “In number” indicates gained chromatin loops that increased in loop number, and “in strength” indicates gained chromatin loops that increased in interaction strength. (**D**) Median distances of “lost” chromatin loops in U87MG cells that were treated with Erastin (10 μM, 72 h). “In number” indicates lost chromatin loops that decreased in loop number, and “in strength” indicates lost chromatin loops that decreased in interaction strength. (**E**) Comparison of gene expression in control U87MG cells (Control_1 and Control_2) and Erastin (10 μM, 72 h)-treated U87MG cells (Erastin_1 and Erastin_2). Heatmap shows clustering of “gained” chromatin loop-associated upregulated genes (log_2_FC > 1; *p_adj_* < 0.05) in control U87MG cells and Erastin (10 μM, 72 h)-treated U87MG cells. (**F**) GO analysis of “gained” chromatin loop-associated upregulated genes (log_2_FC > 1; *p_adj_* < 0.05) in Erastin (10 μM, 72 h)-treated U87MG cells compared to control U87MG cells (dark red color). (**G**) Comparison of gene expression in control U87MG cells (Control_1 and Control_2) and Erastin (10 μM, 72 h)-treated U87MG cells (Erastin_1 and Erastin_2). Heatmap shows clustering of “lost” chromatin loop-associated downregulated genes (log_2_FC ≤ −1 and *p_adj_* < 0.05) in control U87MG cells and Erastin (10 μM, 72 h)-treated U87MG cells. (**H**) GO analysis of “lost” chromatin loop-associated downregulated genes (log_2_FC ≤ −1 and *p_adj_* < 0.05) in Erastin (10 μM, 72 h)-treated U87MG cells compared to control U87MG cells (blue color).

Changes in chromatin loops that were identified by HiChIP were categorized as gained (increased loop number and/or interaction strength) loops or lost (decreased loop number and/or interaction strength) loops in Erastin (10 μM, 72 h)-treated U87MG cells compared with control, and these loops include promoter–promoter (P–P), enhancer–promoter (E–P) and enhancer–enhancer (E–E). In total, 10664 gained loops were identified by HiChIP in ferroptotic glioblastoma cells, including 5563 E–E, 4108 E–P, and 993 P–P (Figure 5A,B). And 15595 lost loops were identified in ferroptotic glioblastoma cells, including 10404 E–E, 4461 E–P, and 730 P–P (Figure 5A,B). In addition, more long-range (>20 kb) chromatin interaction loops were gained or lost in ferroptotic glioblastoma cells (Figure 5C,D), suggesting a significant change of long-range enhancer connectome in ferroptosis. 

Combination analysis of RNA-seq and HiChIP data identified 162 genes that are significantly upregulated (log_2_FC > 1; *p_adj_* < 0.05) in ferroptotic U87MG glioblastoma cells and have gained H3K27ac HiChIP interaction loops (interaction counts ≥ 5) compared to control (Figure 5E). GO analysis of these genes reveals that positive regulation of interleukin-17 production, intrinsic apoptotic signaling pathway in response to DNA damage by p53 class mediator-related biological processes were enriched (Figure 5F), which is associated with ferroptosis [28,29]. At the same time, 302 genes that were significantly downregulated (log_2_FC ≤ −1; *p_adj_* < 0.05) in ferroptotic U87MG glioblastoma cells lost interaction loops (interaction counts ≥ 5) at their loci compared to control (Figure 5G). GO analysis of these downregulated genes showed cell proliferation-associated processes were enriched, like mitotic sister chromatid segregation, mitotic cell cycle, and cell division (Figure 5H). These data suggest transcriptome change in ferroptotic glioblastoma cells is associated with changes in 3D chromatin structures. 

**Figure 6:**
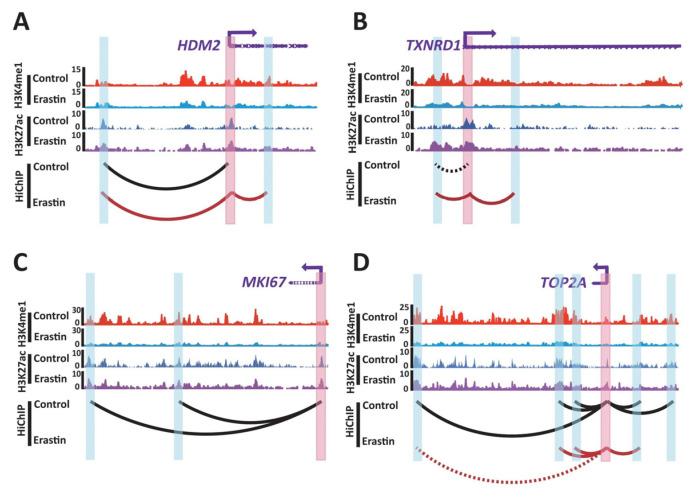
Genes that were regulated by 3D chromatin structures in glioblastoma ferroptosis. (**A**–**D**) H3K4me1 and H3K27ac enrichment for control U87MG cells and U87MG cells that were treated with Erastin (10 μM, 72 h), as well as HiChIP interaction loops between promoters of *HDM2* (**A**), *TXNRD1* (**B**), *MKI67* (**C**), and *TOP2A* (**D**) and enhancers. Promoters are shaded in red; enhancers are shaded in blue. Black curves indicate chromatin interaction loops in control U87MG cells, and red curves indicate chromatin interaction loops in U87MG cells that were treated with Erastin (10 μM, 72 h). Black dashed curves indicate weaker chromatin interactions in control U87MG cells compared to U87MG cells that were treated with Erastin (10 μM, 72 h). Red dashed curves indicate weaker chromatin interactions in U87MG cells that were treated with Erastin (10 μM, 72 h) compared to control. The red track is H3K4me1 ChIP-seq signal from control U87MG cells. The light blue track is H3K4me1 ChIP-seq signal from U87MG cells that were treated with Erastin (10 μM, 72 h). The blue track is H3K27ac ChIP-seq signal from control U87MG cells. The purple track is H3K27ac ChIP-seq signal from U87MG cells that were treated with Erastin (10 μM, 72 h).

The genes that were significantly upregulated in Erastin (10 μM, 72 h)-treated U87MG cells and gained chromatin loops at their respective loci can be ranked by the *p_adj_* value of the transcription fold change; among these, *HDM2* and *TXNRD1* emerged as the top two genes. Western blot was performed to validate the expression levels of *HDM2* and *TXNRD1* in control and U87MG glioblastoma cells that were treated with 10 μM Erastin for 72 h (Figure S2). HDM2 is a negative regulator of tumor suppressor p53 and binds to MDMX to facilitate ferroptosis in cells [30]. HiChIP data showed a new chromatin loop at *HDM2* locus in Erastin (10 μM, 72 h)-treated U87MG cells, and RNA-seq data showed that *HDM2* is significantly upregulated (log_2_FC = 2.44, *p_adj_* = 2.91 × 10^−239^) in Erastin (10 μM, 72 h)-treated U87MG cells, suggesting function of the gained chromatin loop in regulation of *HDM2* (Figure 5E and Figure 6A). *TXNRD1* encodes thioredoxin reductase 1, which regulates cellular redox homeostasis [31]. *TXNRD1* was reported to function in regulating the ferroptosis of liver cancer and chronic myeloid leukemia cells [32,33]. HiChIP data showed a chromatin loop that links a distal enhancer and promoter of *TXNRD1* was strengthened in Erastin (10 μM, 72 h)-treated U87MG cells (Figure 6B), and a new chromatin loop that links an enhancer and promoter of *TXNRD1* was identified in Erastin (10 μM, 72 h)-treated U87MG cells (Figure 6B). In addition, *TXNRD1* was upregulated (log_2_FC = 2.04, *p_adj_* = 1.26 × 10^−216^) compared to control (Figure 5E), which indicated that the gained chromatin loop is related to an increase of *TXNRD1* expression. In addition, the genes that were significantly downregulated in Erastin (10 μM, 72 h)-treated U87MG cells and lost chromatin loops at their respective loci can be ranked by the *p_adj_* value of the transcription fold change; among these, *MKI67* and *TOP2A* emerged as the top two genes. *MKI67* is a marker of cell proliferation that encodes the nuclear protein Ki67 [34]. HiChIP data showed that *MKI67* locus lost two loops that link the *MKI67* promoter and two distal enhancers in Erastin (10 μM, 72 h)-treated U87MG cells, and RNA-seq data showed that *MKI67* is significantly downregulated (log_2_FC = −3.89, *p_adj_* = 0) in Erastin (10 μM, 72 h)-treated U87MG cells (Figure 5G and Figure 6C). *TOP2A* encodes a nuclear enzyme that resolves entanglements and relieves the torsional stress of DNA double strands [35]. *TOP2A* is related to the cell cycle in embryonic stem cells [36]. A chromatin loop that links the *TOP2A* promoter and a distal enhancer was lost in Erastin (10 μM, 72 h)-treated U87MG cells, and another chromatin loop that links the distal enhancer and promoter of *TOP2A* was weakened (Figure 6D). *TOP2A* is also significantly downregulated (log_2_FC = −3.31, *p_adj_* = 0) in Erastin-treated U87MG cells (Figure 5G). These data suggest the association of loss of chromatin loops and downregulation of *MKI67* and *TOP2A* expressions.

## 3. Discussion

**Figure 7:**
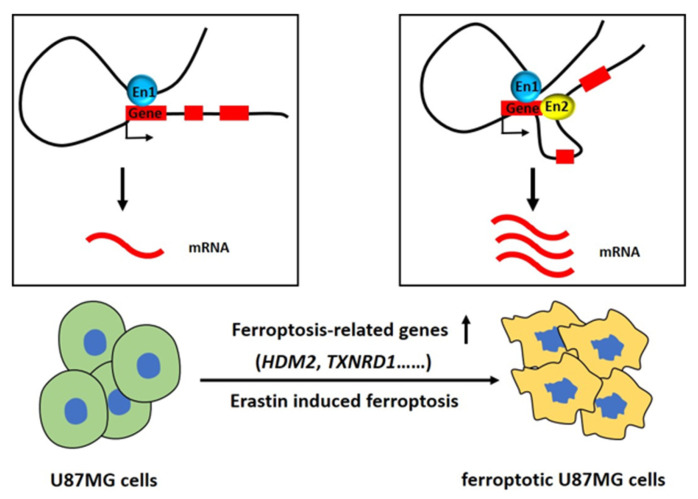
Schematics showed that some ferroptosis-related genes were upregulated and gained chromatin interaction loops between their respective loci and enhancers during Erastin-induced ferroptosis in glioblastoma. En: Enhancer. Blue and yellow rounds represent enhancers, red square represents ferroptosis-related gene, “↓” in the boxes represents transcription, “↑” below the boxes represents up-regulated expression.

The 3D structure of chromatin is reported to play important roles in cell cycle, transcriptional activation, DNA replication, and cell differentiation [37,38,39]. Abnormal 3D chromatin structures and chromatin interactions were found in tumor cells and were reported to be related to cancer progression and other diseases [15,40,41]. Previous studies have shown that CTCF-s activates the expression of *IFI6* by disrupting the 3D spatial conformation of classical CTCF in the *IFI6* region and promoting the interaction between a distal enhancer and the promoter of *IFI6*, leading to apoptosis of HeLa-S3 cells [42]. Recently, several studies have reported that ferroptosis functions in cancer progression and treatment; however, the 3D gene regulation network in ferroptotic cancer cells that mediated by higher-order chromatin structures is not clear. Here in this study, H3K27ac HiChIP was performed and indicated a global change of enhancer-related 3D chromatin structures in ferroptotic glioblastoma cells and revealed that long-range chromatin loops were significantly alternated in ferroptosis. One hundred sixty-two genes that were significantly upregulated in ferroptotic glioblastoma cells correspond to the gain of chromatin loops at their respective loci, and 302 genes that were significantly downregulated in ferroptotic glioblastoma cells correspond to the loss of chromatin loops at their respective loci. These genes include genes that were reported to function in ferroptosis, like *HDM2* and *TXNRD1* (Figure 7). Data in this study suggest a new mechanism by which the change in the 3D chromatin structure in ferroptosis regulates gene expression and functions in glioblastoma cell ferroptosis.

This study focused on enhancer connectome that was induced by 3D chromatin structures. Enhancer–enhancer interactions were important to the regulation of gene expression in biological processes and diseases [43,44]. It has been shown that enhancer interactions in the short distance can ensure high expression of genes through the additive effects [45], and enhancer interactions over the long distance confer functional robustness of gene expression [46]. Recent studies showed that some enhancer interactions can form enhancer hubs, which are connected with key gene promoters and contribute to gene expression in Type 2 diabetes [47]. We noticed 5563 gained enhancer–enhancer interactions in ferroptotic glioblastoma cells (Figure 5A). These new E–E interactions may form enhancer hubs or important regulatory domains and regulate ferroptosis-related genes.

*HDM2* is one of the target genes in the 3D gene regulation network. HDM2 binds to p53 and forms a complex to prevent p53 transcriptional activation. On the other hand, HDM2 is also an E3 ubiquitination ligase, which leads to the degradation of p53 through ubiquitination [48,49]. The HDM2-p53 hub is regulated by different cellular stress signals, and *HDM2* is highly expressed in many types of tumors and is closely related to the proliferation, invasion, apoptosis, and chemotherapy resistance of tumor cells [50,51,52,53,54,55]. Prior studies have shown that HDM2 and MDMX antagonists combined with TMZ have better antitumor activity in experimental animals [56]. In addition, HDM2 was also reported to function in promoting tumor progression in a p53-dependent pathway in glioma cells [48,57,58]. There have been studies showing that HDM2, and likely the HDM2–MDMX complex, are able to increase the sensitivity of glioblastoma cells to ferroptosis [30]. HDM2 antagonist MEL23 can inhibit RSL3-induced cell death in the cells with high expression of *HDM2* but cannot change the sensitivity of the cells with normal expression of *HDM2* to ferroptosis or inhibit RSL3-induced cell death [30]. The function of HDM2 in glioma ferroptosis is related to the E3 ligase activity of the HDM2–MDMX complex. HDM2 and MDMX form complex and regulate lipids by altering the activity of PPARα, which may be a downstream target of E3 ligase activity of HDM2 [30]. Similar mechanisms may also exist in glioblastoma ferroptosis. In our study, HiChIP and RNA-seq results showed that the *HDM2* promoter interacts with an enhancer by a new chromatin loop induced by glioblastoma ferroptosis (Figure 6A), and the expression of *HDM2* is upregulated (Figure 5E), which suggests that change of chromatin loops induced change of *HDM2* expression and then affected glioblastoma ferroptosis. In addition to *HDM2*, *TXNRD1* is another target gene in the 3D gene regulation network; it is an important enzyme with extensive reducing activity in the thioredoxin system [59]. *TXNRD1* is highly expressed in many tumors and is associated with the poor prognosis of tumors [60,61,62,63]. Our results showed that in ferroptotic glioblastoma cells, new enhancer–promoter loops might induce upregulation of *TXNRD1* (Figure 5E and Figure 6B). However, previous studies have shown that the downregulation of *TXNRD1* can promote the production of reactive oxygen species, thereby promoting ferroptosis in tumor cells [64]. We reckon that some rescue pathways may simultaneously work in ferroptotic glioblastoma cells, or complex functions of *TXNRD1* in the regulation of ferroptosis work in different kinds of cells or at different cell stages. 

Ferroptotic cell death is characterized by the accumulation of reactive oxygen species in cells and increased oxidative stress [65], and excessive reactive oxygen species in cells can affect many cell physiological processes, including cell cycle arrest, cell proliferation inhibition, and apoptosis [66,67,68,69]. Several studies have indicated that *MKI67* and *TOP2A* are important genes involved in cell proliferation and cell cycle regulation [70,71,72,73]. HiChIP data in our study indicated attenuated interactions between promoters of *MKI67* and *TOP2A* and enhancers (Figure 6C,D), and RNA-seq data showed downregulation of these two genes (Figure 5G). Downregulation of *MKI67* and *TOP2A* is related to the decrease of cell proliferation [35,73,74,75], which is consistent with the phenotype of ferroptosis. Recent studies show that *MKI67* and *TOP2A* were contained within a network of genes that are upregulated in NK-cell repertoires in patients with neutropenia, which is associated with apoptosis and cell cycle [71]. Though ferroptosis is mechanistically and morphologically different from apoptosis [76], genes known to be involved in apoptosis could also function in ferroptosis, such as *TP53* [77,78]. However, the underlying mechanism of *MKI67* and *TOP2A* involved in ferroptosis is currently not well known. It is not clear whether the downregulation of *MKI67* and *TOP2A* are just induced by increased oxidative stress in ferroptotic glioblastoma cells or whether these two genes also take part in the regulation of ferroptosis. Though bioinformatic analyses suggest that *TOP2A* may be associated with ferroptosis [79,80], further studies are needed to figure out the whole gene regulation network that contains *MKI67* and *TOP2A* in glioblastoma ferroptosis.

Prior studies reported that in p53 wild-type GBM cell line U87MG and p53-mutated GBM cell line U251, P62 plays a dual role in glioma ferroptosis [81], suggestive of different function mechanisms of ferroptosis due to the heterogeneity of glioma cells. In addition, previous studies reported that T98G cells were extremely responsive to Erastin, while U251MG was more resistant to ferroptosis [82]. These studies suggest that the mechanism of ferroptosis may vary in different cell lines. Our study reveals one of the regulation mechanisms in glioblastoma ferroptosis. Further studies are needed to investigate the function of 3D chromatin structures in ferroptosis in view of the heterogeneity of glioma cells.

Temozolomide (TMZ) is a first-line treatment drug for glioma, and a prior study revealed that TMZ inhibits glioma cell growth by inducing ferroptosis by regulating the expression of DMT1 [83], which indicates that the regulation of ferroptosis may affect the drug sensitivity of glioma cells. A recent study designed a nanoscale antibody vector, S-biAb/dEGCG@NPs, which effectively cleared GBM cells in mice by enhancing the effects of ferroptosis and enhancing immune checkpoint blocking (ICB) immunotherapy [84]. A nanodrug, Au (I)-based NIR-II ferrotoxin nanoparticles (TBTP Au NPs), was reported to induce ferroptosis of glioma cells and prolong the survival time of glioma-bearing mice [85]. In addition, ferroptosis was also reported to play an important role in the radiotherapy of glioma [86,87]. Ferroptosis is reported to be associated with various ions [88,89]. Studies of ion metabolism in ferroptosis indicate that ion channels may be a new therapeutic target in glioma [88,89,90,91]. These studies suggest targeting ferroptosis could be an alternative method to improve the poor clinical prognosis of glioma treatment. Our study reveals a new mechanism by which the 3D chromatin structure regulates gene expression in glioblastoma ferroptosis. Thus, specific enhancers or enhancer–promoter interactions may be new therapeutic targets in glioblastoma treatment. Curaxins were reported to target specific 3D chromatin structures in tumors and exert antitumor effects, which indicates the important significance of exploring drug targets based on the 3D structure of chromatin [92,93]. The technology of modification of 3D chromatin structures has been reported [94,95,96], which suggests targeting specific 3D chromatin structures or regulation elements to affect glioblastoma cell ferroptosis could be an alternative method to treat glioblastoma. The findings in this study are helpful in further exploring the mechanism of glioblastoma ferroptosis and discovering new therapeutic targets to treat glioblastoma.

## 4. Materials and Methods

### 4.1. Cell Culture

The glioblastoma cell line U87MG was obtained from the National Infrastructure of Cell Line Resource (Beijing, China). Glioblastoma cells were incubated at 37 °C in 90% humidity and 5% CO_2_ and cultured in DMEM (GIBCO), supplemented with 10% fetal bovine serum (BI) and 1% penicillin/streptomycin (GIBCO). 

### 4.2. MTS Assay

Cell viability was measured using the MTS Assay Kit (Promega, Madison, WI, USA). Briefly, cells were cultured in 96-well plates at a density of 1500 cells/well in a growth medium for 12 h. Cells were subsequently incubated with ferroptosis-inducing compounds Erastin (MCE) at the indicated concentrations in a growth medium for an additional 24 h, 48 h, 72 h, and 96 h. In total, 20 μL (per 100 μL medium) of MTS reagent was added to each well, and absorbance was measured at 490 nm using a microplate reader.

### 4.3. Lipid Peroxidation Assay

The activity level of MDA was assayed following the protocol of the Lipid Peroxidation MDA Assay Kit (Beyotime, Shanghai, China). A total of 200 μL MDA working solution was added to 100 μL of supernatant. The mixture was heated to 100 °C for 15 min. After cooling to room temperature, the mixture was centrifuged at 1000× *g* for 10 min, and 200 μL of supernatant was detected at 532 nm by a microplate reader.

### 4.4. Glutathione and Iron Assay

The intracellular concentration of total GSH was assessed using a GSH Assay Kit (Beyotime, Shanghai, China) according to the manufacturer’s instructions. The content of iron in glioblastoma cells was measured using a Cell Ferrous Iron Colorimetric Assay Kit (Elabscience, Hubei, China) according to the manufacturer’s instructions.

### 4.5. Western Blot

Western blot was performed as previously described [97]. DMSO (control) and Erastin (10 μM, 72 h)-treated U87MG cells were collected and then lysed using RIPA lysis buffer (Solarbio, Beijing, China) with a 1× protease inhibitor cocktail (Sigma Aldrich, St. Louis, MO, USA). Protein concentration was measured using an Enhanced BCA Protein Assay Kit (Beyotime, Shanghai, China) according to manufacturing protocols. The samples were resolved by SDS-PAGE and then transferred to polyvinylidene difluoride (PVDF) membranes (Millipore). The primary antibodies were used as follows: HDM2 (MB67065), TXNRD1 (MB65551) from Bioworld, and β-actin (30101ES50) from YEASEN, and HRP-linked secondary antibodies (sc-516102) from Santa Cruz Biotechnology. Bands were detected by the ImageQuant LAS 4000 system with an enhanced chemiluminescence kit (Thermo Scientific Pierce, Waltham, MA, USA). 

### 4.6. RNA-Seq

Total RNA was extracted from Erastin-induced glioblastoma cells and control cells using TRIzol according to the manufacturer’s protocol. RNA was sequenced by the Novogene. Clean reads were mapped to the Ensemble hg38 human genome using Hisat2 with default parameters. The number of reads mapped to genes was determined with htseq-count [98]. DEGs between treatment and control samples were identified with DEseq2. Genes were considered significantly altered with ∣log_2_ FC∣≥ 1 and *p_adj_* < 0.05. Gene ontology (GO) analysis was carried out using the online tool DAVID (https://david.ncifcrf.gov/ accessed on 10 January 2023). GSEA analysis was performed using GSEA software version 4.3.2 [99].

### 4.7. ChIP-Seq

ChIP-seq assay was performed as previously described [100]. Cells were cross-linked with 1% formaldehyde at RT for 10 min and quenched in 125 mM glycine for 5 min. Cells were resuspended in SDS lysis buffer for 5 min and sonicated to generate DNA fragments averaging 100–500 bp in length. After centrifugation, chromatin fragments were immunoprecipitated with antibodies overnight (H3K27ac, H3K4me1, Abcam, Boston, MA, USA). The precipitated DNA and input DNA were purified and then sequenced. Bowtie2 was used to map ChIP-seq raw reads to the hg38 human reference genome. The MACS2 program was used to call peaks of ChIP-seq data with the corresponding input data as control with default parameters [101,102,103].

### 4.8. HiChIP

HiChIP was performed as previously described [10] using an antibody against H3K27ac (Abcam). Briefly, 1 × 10^7^ cells were cross-linked, and chromatin was digested using *Mbo*I restriction enzyme (NEB). Then, biotin-14-dATP was used to fill in the restriction fragment overhangs and mark the DNA ends. After ligation and sonication, the genomic DNA was incubated with H3K27ac antibody at 4 °C overnight. Immunocomplexes were captured by protein A magnetic beads. The beads were subsequently washed three times with low salt wash buffer, high salt wash buffer, and LiCl wash buffer. DNA was eluted with ddH_2_O and purified with AMPure XP Beads (Beckman Coulter Genetics, Danvers, MA, USA). Biotinylated DNA beads were captured by Streptavidin C-1. QIAseq FX DNA Library Kits (QIAGEN, Hilden, Germany) was used to generate the sequencing library according to the manufacturer’s protocol. The libraries were validated for size distribution of 300–700 bp using AMPure XP beads (Beckman Coulter Genetics, Danvers, MA, USA). The DNA was subjected to 2 × 150 bp paired-end sequencing. The respective paired-end reads were aligned to the hg38 genome by the HiC-Pro pipeline [104]. Default settings were used for the removal of duplicated reads, assignment to *Mbo*I restriction fragments, and filtering for valid interactions. For each cell type, we merged the valid read pairs of individual samples. Hichipper was applied to call loops with default parameters [105]. Reads between samples were normalized to the total number of valid reads. Loops with FDR < 0.05 and supported by at least 5 paired-end tags (PETS) were kept for further analysis. HiC-Pro was used to generate the HiChIP interaction maps [104]. HiChIP interaction maps were then visualized by JuicerBox software version 1.11.08 [106] at 50, 10, and 5 kb resolution as indicated.

### 4.9. Statistical Analyses

Data were analyzed by Student’s *t*-test. ** *p* < 0.01, *** *p* < 0.001.

## 5. Conclusions

In summary, our study shows a new mechanism of ferroptosis that enhancers regulate glioblastoma ferroptosis by 3D chromatin structure-mediated gene regulation networks.

## Data Availability

RNA-seq and DNA-seq raw data used in this study have been deposited in the NCBI Gene Expression Omnibus (GEO) under accession number GSE236253.

## Supplementary Materials

The following supporting information can be downloaded at: https://www.mdpi.com/article/10.3390/ijms241914945/s1.
