# Supplementary material for: Three-Dimensional Gene Regulation Network in Glioblastoma Ferroptosis
Source: Int J Mol Sci. 2023 Oct 6;24(19):14945. doi: 10.3390/ijms241914945 (PMC10574000; doi:10.3390/ijms241914945)
Supplement: Supplementary file 1 [file ijms-24-14945-s001.zip › ijms-2567870-supplementary.pdf]

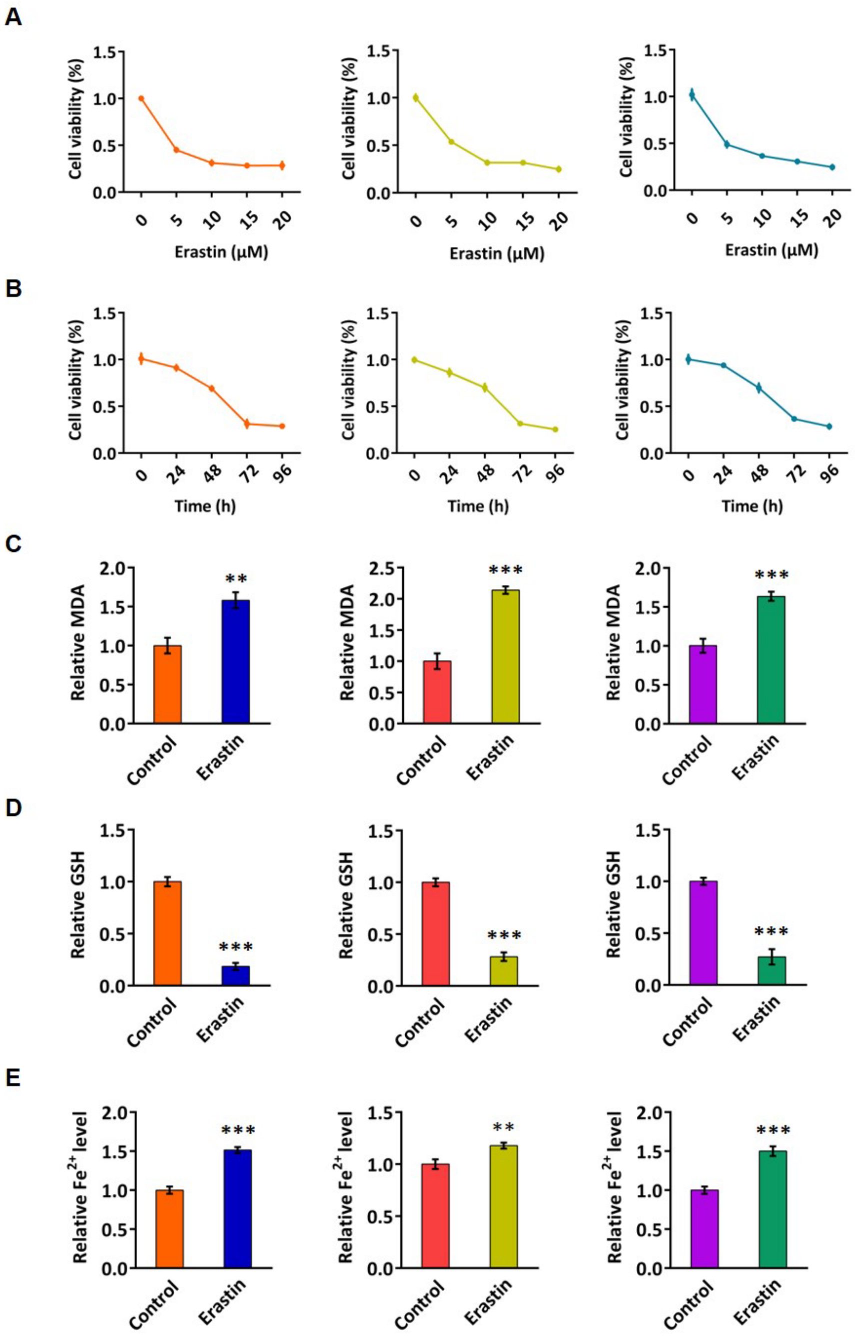

Figure S1. The three replicates data for each experiment in Figure 1. (**A**) Cell viability of U87MG treated with 0, 5, 10, 15 and 20 μM Erastin for 72 h, compared to control. (**B**) Cell viability of U87MG that were treated with 10 μM Erastin for 0 h, 24 h, 48 h, 72 h and 96 h, compared to control. (**C**–**E**) MDA assay, GSH assay and ferrous iron assay indicate increase of MDA (**C**), decrease of GSH (**D**) and accumulation of intracellular Fe^{2+} (**E**) in U87MG cells that were treated with 10 μM Erastin for 72 h. MDA, malondialdehyde; GSH, glutathione. ** $p < 0.01$, *** $p < 0.001$, compared to control.

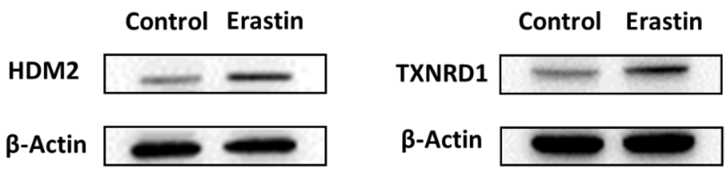

Figure S2. Western blot verification of *HDM2* and *TXNRD1* expression levels in control (DMSO 72h) and U87MG glioblastoma cells that were treated with 10 μM Erastin for 72 h.
